# Time to health-related quality of life score deterioration as a modality of longitudinal analysis for health-related quality of life studies in oncology: do we need RECIST for quality of life to achieve standardization?

**DOI:** 10.1007/s11136-013-0583-6

**Published:** 2013-11-26

**Authors:** Amélie Anota, Zeinab Hamidou, Sophie Paget-Bailly, Benoist Chibaudel, Caroline Bascoul-Mollevi, Pascal Auquier, Virginie Westeel, Frederic Fiteni, Christophe Borg, Franck Bonnetain

**Affiliations:** 1 Quality of Life in Oncology Clinical Research Platform, Besançon, France; 2 Methodological and Quality of Life in Oncology Unit, EA 3181, University Hospital of Besançon, 2 Place Saint-Jacques, 25030 Besançon Cedex, France; 3 Public Health Laboratory, EA 3279, Aix-Marseille University, Marseille, France; 4 Medical Oncology Department, University Hospital Saint-Antoine, Paris, France; 5 Gercor, Clinical Research Group in Oncology, Paris, France; 6 Department of Biostatistics, Regional Cancer Institute, Montpellier, France; 7 Pneumology Department, University Hospital of Besançon, Besançon, France; 8 Medical Oncology Department, University Hospital of Besançon, Besançon, France

**Keywords:** Health-related quality of life, Clinical trials, Oncology, Longitudinal analysis, Time to deterioration

## Abstract

**Purpose:**

Longitudinal analysis of health-related quality of life (HRQoL) remains unstandardized and compromises comparison of results between trials. In oncology, despite available statistical approaches, results are poorly used to change standards of care, mainly due to lack of standardization and the ability to propose clinical meaningful results. In this context, the time to deterioration (TTD) has been proposed as a modality of longitudinal HRQoL analysis for cancer patients. As for tumor response and progression, we propose to develop RECIST criteria for HRQoL.

**Methods:**

Several definitions of TTD are investigated in this paper. We applied this approach in early breast cancer and metastatic pancreatic cancer with a 5-point minimal clinically important difference. In breast cancer, TTD was defined as compared to the baseline score or to the best previous score. In pancreatic cancer (arm 1: gemcitabine with FOLFIRI.3, arm 2: gemcitabine alone), the time until definitive deterioration (TUDD) was investigated with or without death as event.

**Results:**

In the breast cancer study, 381 women were included. The median TTD was influenced by the choice of the reference score. In pancreatic cancer study, 98 patients were enrolled. Patients in Arm 1 presented longer TUDD than those in Arm 2 for most of HRQoL scores. Results of TUDD were slightly different according to the definition of deterioration applied.

**Conclusion:**

Currently, the international ARCAD group supports the idea of developing RECIST for HRQoL in pancreatic and colorectal cancer with liver metastasis, with a view to using HRQoL as a co-primary endpoint along with a tumor parameter.

**Electronic supplementary material:**

The online version of this article (doi:10.1007/s11136-013-0583-6) contains supplementary material, which is available to authorized users.

## Introduction

Although overall survival (OS) is still considered as the “gold standard” for primary endpoints in many oncology studies, most clinical trials now integrate health-related quality of life (HRQoL) as one of the major endpoints to investigate the clinical benefit of new therapeutic strategies for the patient. HRQoL is considered as a second primary endpoint by the American Society of Clinical Oncology and the Food and Drug Administration if no effect of treatment on OS is observed [1–3]. Moreover, since many trials in oncology use so-called surrogate endpoints for OS focusing on tumor parameters, it is of major importance to assess HRQoL in order to characterize the clinical benefit for patients.

Despite this opportunity to achieve comprehensive assessment of HRQoL to support “evidence-based medicine” in oncology, the longitudinal analysis of HRQoL remains unstandardized. This compromises the comparison of results between trials. Moreover, longitudinal results should translate findings into information that decision-makers find understandable and compelling. However, despite the many sophisticated statistical approaches available, results remain underutilized in clinical practice, especially due to a lack of standardization and the inability to propose clinically meaningful results.

Analyses also have to deal with another limiting factor, namely missing data. Patients may not complete the entire HRQoL questionnaire at all planned measurement times. Moreover, patients may drop out before the end of the study, generally due to a deterioration of their health status, or death, as in the palliative setting. Missing data can bias the analysis and interpretation of the results if they depend on the patient’s health status [4–6]. Therefore, there is a need to develop statistical methods that can handle missing data [7–12].

Another challenge of longitudinal HRQoL analysis is to take into account the potential occurrence of a response shift (RS) effect. Indeed, self-assessment of HRQoL is dependent on the patient’s internal standards and the definition of HRQoL used [13–15]. Since patients can adapt to disease and the treatment toxicities, their health and HRQoL expectations may also change over time. These changes result in an RS effect [16]. Sprangers and Schwartz defined RS as “a change in the meaning of one’s self-evaluation of a target construct as a result of the following: (a) a change in the respondent’s internal standards of measurement (i.e., scale recalibration); (b) a change in the respondent’s values (i.e., the importance of component domains constituting the target construct); or (c) a redefinition of the target construct (i.e., reconceptualization)” [17]. Thus, the choice of the reference score to qualify a change such as deterioration is a major concern.

Several methods are used to analyze longitudinal HRQoL data [18–20]. The most widely used is the general linear mixed model (GLMM) [18, 21–23], which is recommended in longitudinal studies with a limited number of follow-up [24]. This method is only adapted when HRQoL assessments are widely spaced and with little amplitude within patients. GLMM can handle the missing data profiles by applying a pattern mixture model [10, 25]. However, these sub-models are rarely applied, mainly because of the complexity of the pattern construction [10, 25–27]. Furthermore, GLMM does not deal with the occurrence of a RS effect.

In the last few years, researchers have started to use models of modern item response theory (IRT) to analyze longitudinal HRQoL data [28]. In contrast to the GLMM, the link between the observed score and the latent trait (e.g., HRQoL) is not linear but logistic. However, these models are rarely used to analyze longitudinal HRQoL data, mainly due to their complexity [29].

Also in recent years, time-to-event models such as the time-to-HRQoL score deterioration (TTD) have been proposed as an approach to the analysis of longitudinal HRQoL in oncology [30, 31]. Both GLMM and TTD rely on the definition of the minimal clinically important difference (MCID) in order to be effective from a clinical point of view. The measure of TTD might be more familiar to clinicians because it is based on Kaplan–Meier survival curves and hazard ratios (HR). As for GLMM, TTD can deal with missing data by making underlying assumptions about whether the missing data reflect a deterioration of the patient’s health status or not. Contrary to GLMM, the TTD method can take into account the occurrence of the RS recalibration component by choosing different reference scores to qualify the deterioration.

TTD cannot be considered as an exclusive method, since the GLMM approach measures different concepts and proposes complementary ways of summarizing HRQoL data. However, if few HRQoL assessments are performed and the interval time between two consecutive assessments is long, then GLMM may be more relevant than the TTD approach. In other cases, the TTD approach may be more suitable than GLMM.

Regarding the TTD approach, the choice of event definition is essential, because it may lead to different results. However, there are currently no recommendations or consensus in this regard, with the result that TTD reflects heterogeneity.

Thus, there is a clear need to investigate and validate several definitions of TTD depending on the following: the cancer context (adjuvant, advanced), reference score, event definitions, MCID, and censoring rules. As for tumor response and progression, one proposition could be to develop “RECIST” criteria (“Response Evaluation Criteria In Solid Tumors”) for HRQoL. This would allow standardization of longitudinal HRQoL analysis using the TTD method, according to the therapeutic situation and the cancer site. Accordingly, several definitions of TTD were investigated and are presented in this paper. We next propose recommendations for the choice of the definition depending on the therapeutic situation. Finally, we report results observed using the TTD approach in early breast cancer and metastatic pancreatic cancer.

## Methods

### Time to deterioration definitions

We propose several definitions of TTD in a HRQoL score according to the therapeutic situation and cancer site. Events can be defined in relation to a reference score, MCID, and missing scores, including death or not. These definitions are summarized in Table 1.

**Table 1:** Summary of the different definitions of time to deterioration (TTD) and time until definitive HRQoL score deterioration (TUDD) investigated

To be considered as events	Reference score			Definitive as compared to			Death	Patients with no baseline	Patients with no follow-up
	Baseline	Best previous score	Previous score	Reference score		Score qualifying the deterioration			
				MCID+^a^	MCID−^b^				
*TTD*									
1	X								
2	X							X	X
3	X						X		
4	X						X	X	X
5		X							
6		X						X	X
7		X					X		
8		X					X	X	X
9			X						
10			X					X	X
11			X				X		
12			X				X	X	X
*TUDD*									
1	X			X					
2	X			X				X	X
3	X			X			X		
4	X			X			X	X	X
5	X				X				
6	X				X			X	X
7	X				X		X		
8	X				X		X	X	X
9	X					X			
10	X					X		X	X
11	X					X	X		
12	X					X	X	X	X
13		X		X					
14		X		X				X	X
15		X		X			X		
16		X		X			X	X	X
17		X			X				
18		X			X			X	X
19		X			X		X		
20		X			X		X	X	X
21		X				X			
22		X				X		X	X
23		X				X	X		
24		X				X	X	X	X
25			X	X					
26			X	X				X	X
27			X	X			X		
28			X	X			X	X	X
29			X		X				
30			X		X			X	X
31			X		X		X		
32			X		X		X	X	X
33			X			X			
34			X			X		X	X
35			X			X	X		
36			X			X	X	X	X

A cross (X) indicates the retained definition and the corresponding events

^a^MCID+ deterioration with no further improvement as compared to the reference score (definition of Bonnetain et al.)

^b^MCID− definitive deterioration if deterioration observed at all time points following the initial deterioration

1. Core definitions with respect to the MCID

The most intuitive definition for TTD is the time from inclusion–randomization in the study to

**Fig. 1:**
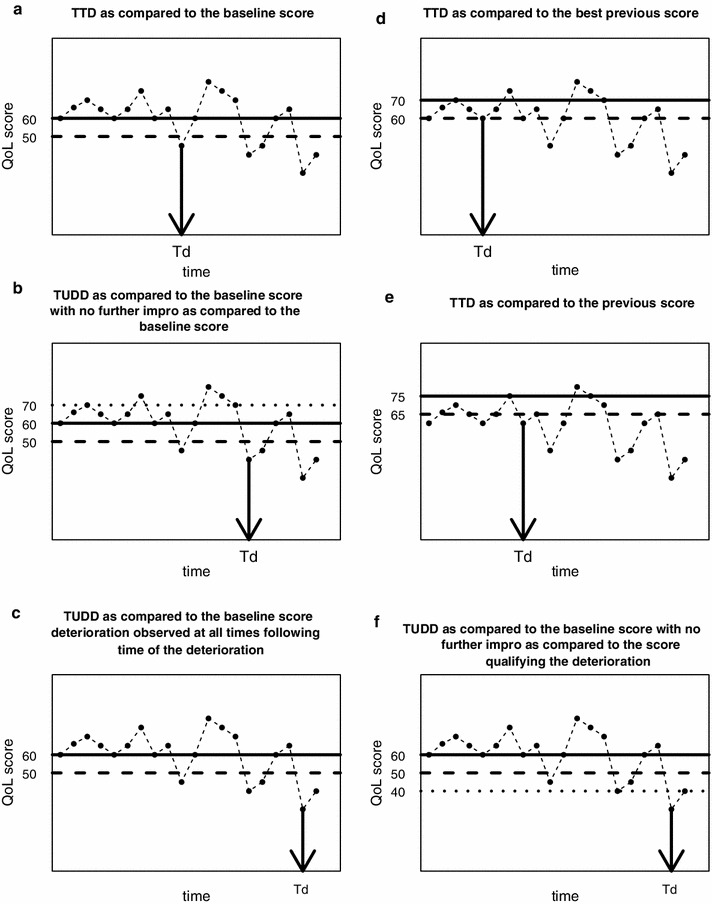
Illustration of time to deterioration (Td) using different definitions with a 10-point MCID for one patient and for a health-related quality of life score (QoL) in which a deterioration corresponds to a decrease in the score. The *solid line* corresponds to the value of the reference score at time Td. The *dashed line* corresponds to the threshold to observe deterioration as compared to the reference score at time Td. The *dotted line* corresponds to the threshold to observe a definitive deterioration as compared to the reference score at time Td
- Patients with no deterioration before their dropout are censored at the time of the last follow-up or the last HRQoL assessment.

- a first deterioration of at least one MCID unit as compared to the baseline score [31] (Fig. 1a).

This definition corresponds to definition TTD#1 in Table 1.

According to the scoring algorithm of the HRQoL dimension, the deterioration corresponds to an increase or decrease in at least one MCID unit of the score as compared to the baseline score. The MCID may vary depending on the instruments and cancer sites under consideration.

The deterioration observed can be definitive or not. In the palliative setting, Bonnetain et al. have previously defined the time until definitive HRQoL score deterioration (TUDD) as the time from inclusion in the study to a first deterioration of at least one MCID unit as compared to the baseline score:

- with no further improvement of more than one MCID unit as compared to the baseline score (Fig. 1b).
- or if the patient dropped out after deterioration, resulting in missing data.

This corresponds to the definition TUDD#1 in Table 1.

An alternative for defining TUDD is to consider that the first deterioration of at least one MCID unit observed at time T is definitive:

- if the deterioration of at least one MCID unit as compared to the baseline score is also observed at all time points after time T (Fig. 1c).
- or if the patient dropped out after deterioration, resulting in missing data.

This second definition of TUDD corresponds to definition TUDD#5 of Table 1.

2) Alternatives for defining the reference score

The concept of deterioration requires a reference score relative to which the deterioration may be quantified. In the definitions described here, the reference score is the baseline score. However, the reference score could also be defined in other ways. For example,

- the best previous HRQoL score. Figure 1d illustrates the TTD with a 10-point MCID as compared to the best previous HRQoL score for one patient (TTD#5 in Table 1) or
- the previous HRQoL score. Figure 1e illustrates the TTD with a 10-point MCID for one patient with the previous score (i.e., “immediately preceding score”) as the reference score (TTD#9 in Table 1).

Moreover, for definitive deterioration, the deterioration observed at time T can be considered definitive:

- as compared to the reference score (baseline score, previous score, or best previous score) or
- as compared to the score qualifying the deterioration (i.e., the score obtained at time T). In that case, the score qualifying the deterioration at time T becomes the reference score (TUDD#9). Figure 1f. illustrates the TUDD as compared to the baseline score with no further improvement as compared to the score qualifying the deterioration for one patient.

3) Missing data issues

Intermittent missing data are ignored in the TTD approach, which goes on the assumption that HRQoL level remains unchanged since the last available HRQoL assessment. Moreover, patients with no baseline HRQoL score or with no follow-up score are usually excluded from longitudinal analysis. However, these patients can be included in the analysis and censored at baseline or just after baseline. Depending on the therapeutic situation, sensitivity analysis can be performed considering these patients to be deteriorating since baseline. For example, definition TUDD#2 in Table 1 corresponds to TUDD as compared to the baseline score, according to the definition of Bonnetain et al., including patients with no baseline HRQoL score or with no follow-up score as events.

4) Death as an event

All-cause death can be considered as an event if the patient did not experience deterioration before death. These supplementary events (death, no follow-up) will be addressed in the case of TUDD. In this way, TUDD or death could be redefined as “HRQoL deterioration-free survival.” For example, definition TUDD#3 in Table 1 corresponds to TUDD as compared to the baseline score according to the definition of Bonnetain et al., or death.

5) Response shift issue

Patients’ internal standards can change over time, reflecting the recalibration component of RS. An alternative way to take into account the occurrence of the recalibration component of RS could be to consider the reference score as the best previous HRQoL score, or the previous (immediately preceding) HRQoL score but not the baseline sore. The value of these scores can change over time according to the patient’s experience of treatment and disease course.

6) Multidimensional definition

We can study the deterioration of one given HRQoL score, or the deterioration of at least one HRQoL dimension among the set of all dimensions. For example, we can study deterioration of at least one dimension of a multidimensional questionnaire. In the case of a multidimensional definition, the event time corresponds to the first deterioration observed, irrespective of which HRQoL score is affected. In this situation, competitive risks should be taken into account. This multidimensional definition has the advantage of increasing the statistical power and may be relevant if the treatment is expected to have a similar effect on all the HRQoL dimensions retained.

As TTD analyses count as survival analyses, the TTD estimation can be calculated using the Kaplan–Meier or actuarial method and described using median and 95 % confidence interval (CI). The Kaplan–Meier survival curve is defined as the probability of surviving in a given length of time while considering time in many small intervals. This method is based on the intuitive idea that being alive at time T naturally requires the subject to be alive just before time T, and not to die at time T [32]. Contrary to the Kaplan–Meier method, in the actuarial method, probabilities are estimated for fixed time intervals, not determined by the date of observed death. Both methods can handle the presence of censored data, i.e., patients are still alive at the end of the study.

In time to deterioration (TTD) analyses, the event is “the HRQoL score deterioration.” The Kaplan–Meier estimation is given by the following formula:

$$S\left( { t} \right) = \mathop \prod \limits_{{t_{i} \le t}} \frac{{n_{i} - m_{i} }}{{n_{i} }}$$

where $n_{i} = n_{i - 1} - m_{i - 1} - c_{i - 1}$and *n*_*i*_ is the number of subject at risk at time *T*_*i*_, i.e., the number of patients still in the study and who do not present a deterioration until time *T*_*i*−1_, *m*_i_ is the number of events observed at time *T*_*i*_, i.e., the number of patients experiencing a HRQoL score deterioration at time *T*_*i*_, and *c*_*i*_ is the number of censored patients at time *T*_*i*_, i.e., the number of patients who dropped out at time *T*_*i*_ and who did not experienced a HRQoL deterioration before.

TTD can then be compared according to treatment arm using the log-rank test and univariate Cox analyses to produce a HR with 95 % CI. Multivariate Cox regression can be applied to identify independent factors associated with TTD.

**Fig. 2:**
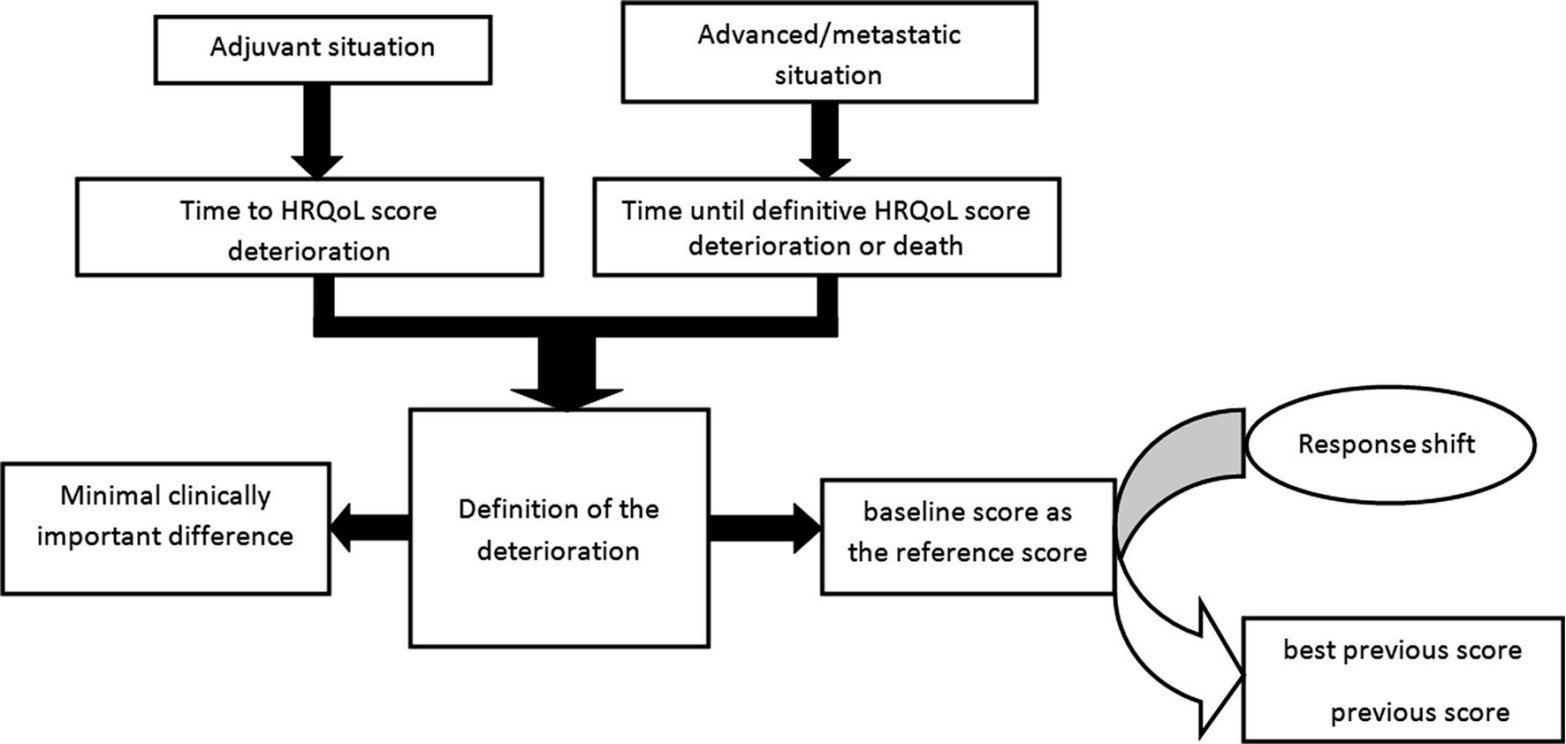
Decision-making flowchart according to the therapeutic situation

In Fig. 2, we propose a decision-making flowchart. In the adjuvant setting, we recommend using the TTD; and in the advanced or metastatic setting, we recommend using the TUDD, with or without death from all causes as an event. Indeed, it is intuitive that in the adjuvant setting, deterioration is expected not to be definitive, because the patient could conceivably survive the cancer. Moreover, cancer survivors can experience an improvement of their HRQoL. In contrast, in the advanced or metastatic setting, a definitive deterioration is more relevant, reflecting the deterioration of the patient’s health status, which is stable over time. Furthermore, the time between deterioration and death is often short for these patients [30]. The definition of the deterioration is based on both the threshold for the MCID, and the definition chosen for the reference score. Thus, if no RS effect occurs, the baseline score can be kept as the reference score in the TTD analysis. If a RS is likely to occur, we recommend using the best previous score or the previous score as the reference score in the TTD analysis.

### Health-related quality of life studies

In this section, we report TTD analyses performed in two studies as an illustration, namely early breast cancer and metastatic pancreatic cancer. In the breast cancer study, since it is an adjuvant setting, we retained the TTD approach and studied the impact of RS on TTD using changing score as the reference score, i.e., the best previous score. In the metastatic pancreatic cancer study, as it is a metastatic setting, we retained the TUDD approach, integrating death (or not) as event. We also took into account informative missing data.

#### Time to deterioration in early breast cancer

A prospective, multicenter, randomized, cohort study including all women hospitalized for the diagnosis or treatment of first primary breast cancer or for a suspicion of breast cancer was performed in French hospitals between February 2006 and February 2008. All participants gave written informed consent, and the local ethics committee approved the study protocol. The complete design of this study has previously been described elsewhere [33].

HRQoL was evaluated using the EORTC cancer-specific questionnaire QLQ-C30 [34] and its breast cancer module QLQ-BR23 [35]. These were administered at inclusion, at discharge following initial hospitalization, as well as at three and 6 months after inclusion. The QLQ-C30 and its breast cancer module BR23 are validated tools to assess HRQoL in cancer, specifically in breast cancer [34, 35].

The QLQ-C30 includes 30 items and measures five functional scales (physical, role, emotional, cognitive, and social functioning), global health status (GHS), financial difficulties, and eight symptom scales (fatigue, nausea and vomiting, pain, dyspnea, insomnia, appetite loss, constipation, and diarrhea) [34].

The BR23 module includes 23 items that generate four functional scales (body image, sexual functioning, sexual enjoyment, and future perspective) and four symptom scales (systemic therapy side effects, breast symptoms, arm symptoms, and upset caused by hair loss) [35].

The occurrence of a RS effect has already been demonstrated in early breast cancer patients [33, 36] and particularly in this study [31, 33]. Thus, two definitions of TTD were investigated using a 5-point MCID: The first definition was TTD with the baseline score as the reference score [31]. The second was TTD with the best previous score as the reference score. Patients with at least one HRQoL score were included in the TTD analysis. Patients with no follow-up HRQoL score were censored just after baseline. Patients with no baseline score were censored at baseline.

TTD curves were calculated using the Kaplan–Meier estimation and described using median and 95 % CI.

#### Time until definitive deterioration in metastatic pancreatic cancer

This study was a multicenter, randomized, open phase II trial conducted in 11 French centers between October 2007 and May 2011. Randomization 1:1 was done using the minimization technique with stratification according to center, performance status (0 vs. 1), and the number of metastatic sites (one vs. more than one).

Inclusion criteria were as follows: histologically or cytologically proven metastatic pancreatic adenocarcinoma, no previous chemotherapy, no previous radiotherapy, and WHO performance status <2.

Exclusion criteria were bile duct adenocarcinoma, ampulloma, and history of another major cancer.

All patients were fully informed of the study and provided written informed consent. The protocol was approved by the ethics committee.

Patients were randomly assigned to receive alternately FOLFIRI 3 every 14 days for 2 months (i.e., 4 courses per cycle), followed by gemcitabine, 6 courses at days 1, 8, 15, 29, 36, and 43 per cycle (Arm 1) or gemcitabine alone (Arm 2). FOLFIRI 3 is a chemotherapeutic regimen combining 5-fluorouracil, folinic acid, and irinotecan.

HRQoL was evaluated using the QLQ-C30 questionnaire [34] at inclusion and every 2 months until the end of the study or death.

The TUDD was defined as the TUDD with a 5-point MCID as compared to the baseline score, with no further improvement of more than 5 points [30]. Patients with at least one HRQoL score were included in the TUDD analysis. Patients with no baseline score were censored at baseline. Patients with no follow-up measures were censored just after baseline. Sensitivity analyses were conducted, first considering death as an event and then simultaneously considering death and no follow-up as events. TUDD analyses including death as an event are referred to “HRQoL deterioration-free survival” analyses.

TUDD curves were calculated using the Kaplan–Meier method and described using median and 95 % CI. TUDD was compared between treatment arms using the log-rank test and univariate HR with 95 % CI.

For both studies, variables collected at baseline are described as means and standard deviations (SD) for continuous variables and number (percentage) for qualitative variables. The percentage of missing data is also provided. The number of HRQoL questionnaires completed at each measurement time is reported. Scores were generated according to the EORTC scoring manual [37]. These scores vary from 0 (worst) to 100 (best) for the functional dimensions and GHS, and from 0 (best) to 100 (worst) for the symptom dimensions.

All analyses were performed with R software [38].

## Results

### Breast cancer

Between February 2006 and February 2008, 381 patients were included in the four participating centers. Mean age was 58.4 (SD = 11) years. Complete clinical and pathologic characteristics of the population are given in supplementary Table A.

At baseline, 359 (94 %) patients had at least one HRQoL score, 343 (90 %) at discharge following initial hospitalization, 340 (89 %) at three months, and 321 (84 %) at 6 months.

Results of the TTD analyses are summarized in Table 2.

**Table 2:** Results of the Kaplan–Meier estimation of the time to deterioration (TTD) for each QLQ-C30 score and QLQ-BR23 score with the baseline score or the best previous score as the reference score regarding breast cancer study (study #1)

	TTD baseline score		TTD best previous score	
	*n* (events)	Median in months (95 % CI)	*n* (events)	Median in months (95 % CI)
*QLQ*-*C30*				
Global health status	376 (224)	3.0 (2.8–3.0)	376 (263)	3.0 (2.9–3.0)
Physical functioning	376 (255)	0.2 (0.2–2.8)	376 (290)	0.4 (0.2–2.9)
Role functioning	375 (235)	3.0 (3.0–3.0)	375 (262)	3.0 (3.0–3.0)
Emotional functioning	377 (153)	6.1 (6.0–NA)	377 (232)	5.6 (3.2–5.9)
Social functioning	377 (193)	3.1 (3.0–5.9)	377 (221)	3.1 (3.0–5.4)
Cognitive functioning	377 (160)	6.1 (5.4–NA)	377 (197)	3.5 (3.2–6.0)
Fatigue	374 (248)	2.7 (0.2–3.0)	374 (282)	2.9 (0.4–3.0)
Pain	377 (234)	3.0 (0.6–3.0)	377 (268)	4.0 (2.8–3.0)
Nausea and vomiting	375 (123)	7.0 (6.1–NA)	375 (139)	7.0 (6.1–NA)
Dyspnea	375 (126)	6.2 (6.1–NA)	375 (164)	6.1 (6.0–6.2)
Insomnia	374 (141)	6.1 (6.0–NA)	374 (194)	6.0 (5.7–6.0)
Appetite loss	375 (106)	NA (6.3–NA)	375 (124)	6.5 (6.3–NA)
Constipation	377 (147)	6.2 (6.0–NA)	377 (173)	6.0 (5.9–6.4)
Diarrhea	375 (59)	NA (6.5–NA)	375 (81)	6.5 (6.4–NA)
Financial difficulties	376 (70)	NA (6.4–NA)	376 (78)	NA (6.4–NA)
*QLQ*-*BR23*				
Body image	376 (207)	3.0 (3.0–3.1)	376 (236)	3.0 (3.0–3.2)
Sexual functioning	354 (71)	6.4 (6.3–NA)	354 (118)	6.2 (6.1–6.4)
Sexual enjoyment	224 (21)	7.4 (6.4–NA)	224 (45)	6.4 (6.2–NA)
Future perspective	375 (90)	7.0 (6.6–NA)	375 (165)	6.1 (6.0–6.1)
Systemic therapy side effects	376 (194)	3.1 (3.0–3.4)	375 (233)	3.1 (3.0–3.2)
Breast symptoms	375 (228)	0.2 (0.2–2.8)	375 (284)	3.0 (2.8–3.0)
Arm symptoms	375 (214)	2.9 (0.4–3.1)	375 (247)	6.0 (3.6–6.0)
Upset by hair loss	194 (16)	3.3 (3.1–NA)	194 (38)	6.3 (6.2–NA)

**Fig. 3:**
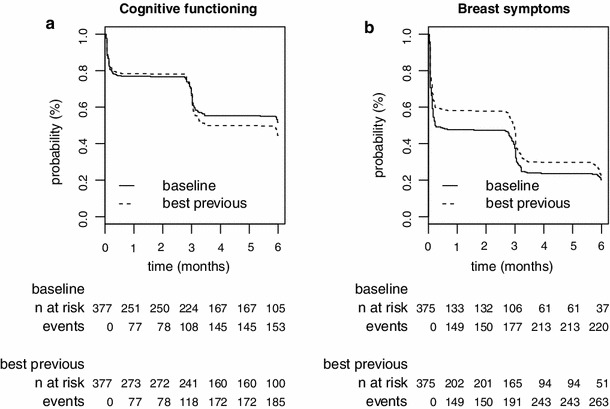
Time to HRQoL score deterioration curves with a 5-point MCID for breast cancer (study #1) with baseline score or best previous score as the reference score for cognitive functioning (CF) (*panel A*), and breast symptoms (BS) (*panel B*)

Among the 377 patients included with at least one cognitive functioning score, 160 and 197 patients presented deterioration of cognitive function as compared to the baseline score and the best previous score, respectively. The median TTD decreased from 6.1 months [5.4–NA] when baseline was the reference score to 3.5 [3.2–6.0] when the reference was the best previous score (Fig. 3a).

Among the 375 patients included with at least one breast symptoms score, 228 and 284 patients presented breast symptom deterioration as compared to the baseline score and the best previous score, respectively. The median TTD increased from 0.2 months [0.2–2.8] when recalibration was not taken into account to 2.8 [2.8–3.0] when it was taken into account (Fig. 3b).

Among the 375 patients included with at least one arm symptoms score, 214 and 247 patients presented arm symptoms deterioration as compared to the baseline score and to the best previous score, respectively. The median TTD increased from 2.9 months [0.4–3.1] when recalibration was not taken into account to 6.0 [3.6–6.0] when it was.

### Pancreatic cancer

Between October 2007 and May 2011, 98 patients were enrolled in 10 French centers. Mean age was 62 years (SD = 8.4). The baseline characteristics of the patients are summarized in supplementary Table B.

At baseline, 34 patients (69.4 %) completed the QLQ-C30 questionnaire in Arm 1 (gemcitabine + FOLFIRI 3) and 30 patients (61.2 %) in Arm 2 (gemcitabine alone) (supplementary Table C).

**Fig. 4:**
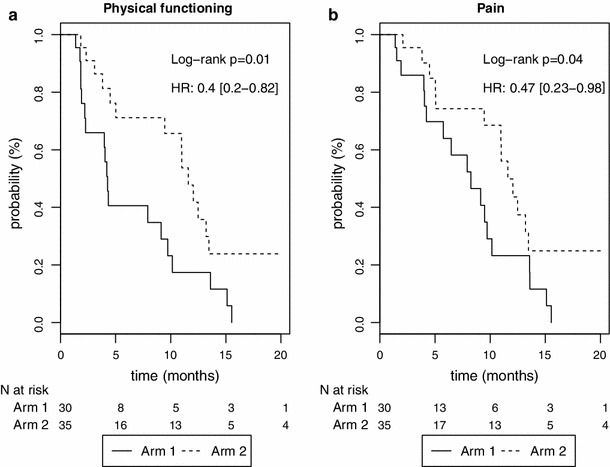
Kaplan–Meier survival curves for the time until definitive deterioration or death for the pancreatic cancer study (study #2)

The TUDD as compared to the baseline score with a 5-point MCID or death was retained for the primary analysis. The Kaplan–Meier curves showing TUDD for the physical functioning and pain scales are shown in Fig. 4.

Patients in Arm 1 (gemcitabine + FOLFIRI 3) seem to present a longer TUDD than those in Arm 2 (gemcitabine alone) for each HRQoL score (Table 3).

**Table 3:** Results of the Kaplan–Meier estimation of the time until definitive deterioration (TUDD) for each QLQ-C30 score^1^ and comparison between arms regarding pancreatic cancer study (study #2)

		TUDD baseline				TUDD baseline or death				TUDD baseline or death or no follow-up			
		n (events)	Median (CI 95 %)	Log-rank	HR (CI 95 %)	n (events)	Median (CI 95 %)	Log-rank	HR (CI 95 %)	n (events)	Median (CI 95 %)	Log-rank	HR (CI 95 %)
GHS	Gemcitabine alone	30 (6)	4.34 (2.2–NA)		1	30 (18)	7.92 (4.21–13.6)		1	30 (25)	4.27 (2.2–9.72)		1
	Gemcitabine + folfiri.3	33 (6)	3.22 (1.97–NA)	0.82	1.14 (0.37–3.54)	33 (16)	9.46 (3.81–NA)	0.45	0.77 (0.38–1.55)	33 (28)	3.22 (1.15–12.06)	0.95	1.02 (0.58–1.76)
PF	Gemcitabine alone	30 (9)	2.27 (1.91–NA)		1	30 (19)	4.27 (2.27–10.15)		1	30 (26)	3.98 (1.84–9.13)		1
	Gemcitabine + folfiri.3	35 (5)	12.06 (11.6–NA)	0.02	0.23 (0.06–0.85)	35 (17)	11.6 (9.46–26.25)	0.01	0.4 (0.2–0.82)	35 (30)	4.5 (0.03–12.06)	0.21	0.7 (0.41–1.22)
RF	Gemcitabine alone	30 (6)	4.27 (1.91–NA)		1	30 (18)	6.47 (4.04–13.57)		1	30 (25)	4.04 (1.84–9.72)		1
	Gemcitabine + folfiri.3	35 (5)	12.06 (7.36–NA)	0.17	0.39 (0.1–1.57)	35 (16)	11.01 (7.36–22.57)	0.15	0.6 (0.29–1.21)	35 (29)	4.5 (0.03–12.06)	0.70	0.9 (0.52–1.56)
EF	Gemcitabine alone	30 (9)	4.27 (2.2–NA)		1	30 (19)	5.75 (4.04–9.72)		1	30 (26)	4.21 (1.91–8.25)		1
	Gemcitabine + folfiri.3	33 (8)	5.06 (1.91–NA)	0.60	0.76 (0.28–2.07)	33 (18)	11.01 (3.81–22.57)	0.05	0.5 (0.25–1.02)	33 (29)	3.68 (0.92–12.48)	0.32	0.75 (0.43–1.32)
CF	Gemcitabine alone	29 (5)	4.34 (4.01–NA)		1	29 (17)	8.25 (4.27–13.57)		1	29 (23)	4.34 (4.01–9.72)		1
	Gemcitabine + folfiri.3	32 (7)	6.01 (3.81–NA)	0.95	0.97 (0.29–3.26)	32 (17)	9.46 (5.03–13.21)	0.69	0.87 (0.43–1.73)	32 (28)	3.81 (1.84–10.97)	0.70	1.12 (0.64–1.96)
SF	Gemcitabine alone	30 (7)	4.27 (2.2–NA)		1	30 (18)	7.92 (4.04–13.57)		1	30 (25)	4.04 (1.91–9.72)		1
	Gemcitabine + folfiri.3	33 (8)	3.22 (2.07–NA)	0.95	1.03 (0.36–2.95)	33 (19)	9.46 (3.22–13.47)	0.37	0.73 (0.37–1.45)	33 (30)	3.09 (1.15–11.01)	0.85	0.95 (0.55–1.64)
FA	Gemcitabine alone	30 (6)	4.34 (2.2–NA)		1	30 (18)	7.92 (4.21–13.57)		1	30 (25)	4.21 (1.91–9.72)		1
	Gemcitabine + folfiri.3	35 (6)	11.6 (8.57–NA)	0.15	0.38 (0.09–1.52)	35 (17)	11.01 (8.57–13.47)	0.13	0.59 (0.3–1.18)	35 (30)	4.5 (0.03–11.6)	0.67	0.89 (0.51–1.53)
NV	Gemcitabine alone	30 (5)	NA (2.33–NA)		1	30 (18)	8.25 (4.04–13.57)		1	30 (25)	6.47 (2.33–9.49)		1
	Gemcitabine + folfiri.3	33 (3)	NA (NA–NA)	0.40	0.54 (0.13–2.29)	33 (14)	12.48 (9.46–NA)	0.04	0.47 (0.22–0.99)	33 (25)	5.03 (1.81–13.21)	0.30	0.74 (0.42–1.31)
PA	Gemcitabine alone	30 (4)	5.75 (5.75–NA)		1	30 (18)	8.25 (5.75–13.57)		1	30 (25)	5.75 (3.98–9.72)		1
	Gemcitabine + folfiri.3	35 (4)	12.06 (11.6–NA)	0.16	0.31 (0.05–1.76)	35 (16)	11.6 (10.97–NA)	0.04	0.47 (0.23–0.98)	35 (29)	5.04 (0.03–12.06)	0.41	0.79 (0.45–1.38)
DY	Gemcitabine alone	30 (4)	4.86 (2.33–NA)		1	30 (17)	8.69 (4.86–13.6)		1	30 (24)	6.47 (2.33–9.72)		1
	Gemcitabine + folfiri.3	35 (3)	15.64 (NA–NA)	0.15	0.3 (0.05–1.69)	35 (15)	12.48 (9.46–NA)	0.02	0.41 (0.19–0.89)	35 (28)	5.03 (0.03–13.21)	0.38	0.76 (0.43–1.34)
In	Gemcitabine alone	30 (8)	4.37 (1.91–NA)		1	30 (19)	5.75 (3.98–9.2)		1	30 (26)	4.04 (1.84–8.25)		1
	Gemcitabine + folfiri.3	35 (2)	12.06 (12.06–NA)	0.01	0.07 (0.01–0.56)	35 (13)	12.06 (10.97–NA)	<0.01	0.24 (0.11–0.54)	35 (26)	5.03 (0.03–13.21)	0.04	0.56 (0.31–1)
AP	Gemcitabine alone	30 (5)	4.83 (4.27–NA)		1	30 (19)	7.92 (4.27–13.57)		1	30 (26)	4.83 (3.98–9.49)		1
	Gemcitabine + folfiri.3	35 (4)	12.06 (12.06–NA)	0.14	0.31 (0.06–1.62)	35 (15)	12.06 (10.97–NA)	0.04	0.47 (0.23–0.98)	35 (28)	5.03 (0.03–12.48)	0.41	0.79 (0.45–1.38)

^1^The QLQ-C30 measures five functional scales (*PF* physical functioning, *RF* role functioning, *EF* emotional functioning, *CF* cognitive functioning, *SF* social functioning), *GHS* global health status, and nine symptom scales (*FA* fatigue, *NV* nausea and vomiting, *PA* pain, *DY* dyspnea, *In* insomnia, *Ap* appetite loss, constipation, diarrhea, and financial difficulties). Results for constipation, diarrhea, and financial difficulties are not shown

Whatever the definition applied, patients in Arm 1 (gemcitabine + FOLFIRI 3) presented a longer TUDD of insomnia than those of Arm 2 (gemcitabine alone) with HR < 1.

Regarding TUDD definitions integrating death or not, patients in Arm 1 (gemcitabine + FOLFIRI 3) presented a longer TUDD than those in Arm 2 (gemcitabine alone) for physical functioning, but this trend was no longer significant when we considered patients with no follow-up as having deteriorated at baseline.

## Discussion

Definitions of deterioration applied in this paper, such as TTD compared to baseline score in breast cancer, and TUDD according to the definition of Bonnetain et al. in the pancreatic cancer study, have also been applied in other studies [39, 40]. This demonstrates the didactic nature of this approach.

Different definitions of TTD have been proposed and investigated in this paper. According to the definition applied, results can change and this precludes comparison of results between oncology clinical trials. The multiplicity of possible event definitions is a limitation of TTD analysis, as it can change the conclusions drawn from the same study. For this reason, it is essential to achieve a consensus. Moreover, if interval estimation of survival analysis is used, the “real” deterioration time is unknown, and as a result, the TTD will be overestimated, but biological markers such as progression-free survival also use this estimation method. An alternative is under investigation, for example, with patients completing the HRQoL questionnaire when they perceive a change.

In this paper, we report the results of TTD analyses according to different therapeutic situations (adjuvant or metastatic) and cancer sites (breast and pancreatic cancers). The impact of some challenges of longitudinal HRQoL analysis on TTD is also studied, namely occurrence of RS in breast cancer study and missing data in pancreatic cancer. We adjusted the definition of deterioration and the choice of the reference score according to the problem being studied.

In the breast cancer study, we noted that the choice of the reference score impacted on the median TTD. When the best previous score was used as the reference, rather than the baseline score, the median TTD of cognitive functioning decreased while that of the breast and arm symptoms increased. The median TTD is sensitive to the choice of reference score. One limitation of this study is the number of HRQoL assessments. Only four assessments of HRQoL during the study were planned. In the pancreatic cancer study, results were slightly different according to the definition applied. Regarding TUDD definitions integrating death or not, patients in Arm 1 (gemcitabine + FOLFIRI 3) presented a significantly longer TUDD than those of Arm 2 (gemcitabine alone) for physical functioning, but this trend was no longer significant when we considered patients with no follow-up as deteriorated at baseline.

In early breast cancer (study #1), the TTD definition applied, using the best previous score as the reference, has the advantage of taking into account the occurrence of the recalibration component of RS. The occurrence of short-term recalibration in this study was previously demonstrated [33]; thus, we had to adjust the method of longitudinal analysis according to the change in the patients’ internal standards. Different methods of assessing RS exist [41–43]. However, the challenge is to take into account the occurrence of the RS effect in longitudinal analysis in order to estimate the true change. The “then-test” method, which assesses patients’ pretest HRQoL levels retrospectively, is the most popular method to assess RS [44]. However, this method is time-consuming, and given its retrospective nature, the then-test is susceptible to recall bias [45]. The TTD approach has the advantage of taking recalibration into account without additional questionnaires, by using changing scores as a reference. Currently, few longitudinal methods can integrate the occurrence of a RS effect. Structural equation modeling can separate true change from RS effect [42, 46]. However, due to the complexity of this method, it is difficult to propose a simple interpretation of these models to clinicians.

The TTD approach is suitable for different therapeutic situations. Indeed, using the pancreatic cancer study, we integrated the metastatic component as a definitive deterioration with death as an event.

Many definitions of deterioration have been proposed in this paper. The choice of the event definition is essential, because it may induce different results. However, there is currently no recommendation or consensus on this point. Consequently, TTD reflects heterogeneity. In the adjuvant setting, we thus recommend using the TTD; and in the advanced or metastatic setting, we recommend using the TUDD with or without death as an event. The baseline score could be considered as the reference score if there is no evidence of a RS effect. If a RS is likely to occur, we recommend using the best previous score or the previous score as the reference score in the TTD analysis.

As in other statistical methods for longitudinal analysis, the TTD approach can handle the occurrence of missing data by making some underlying assumptions, either by considering that the HRQoL level is constant for intermittent missing data, or by considering the missing HRQoL score as revealing the deterioration of the patient’s health status. Few statistical methods handle missing data in longitudinal studies of HRQoL, and these methods are rarely applied due to their complexity. Pattern mixture models have been proposed to analyze longitudinal HRQoL with missing data [10, 25]. However, the number of patterns may be considerable and makes difficult the estimation of the model parameters for each plan. In this way, the TTD approach seems to be more appropriate than GLMM with pattern mixture for studies with many HRQoL assessments, although these two approaches measure different concepts, and thus, TTD cannot be a substitute for GLMM. In the pancreatic cancer study, we considered patients with no follow-up measure as having deteriorated since baseline. Further research is needed to take into account missing data profiles in TTD analyses. We are currently developing a method to use in conjunction with TTD to take into account missing not-at-random data using a method derived from a propensity score.

Results of TTD analysis could be more suitable than GLMM for clinicians, who are familiar with survival analysis, with HR, and log-rank test. However, both GLMM and TTD rely on the definition of MCID to be effective from a clinical point of view. Thus, these methods share the same limitation deriving from the lack of consensus around the MCID definition. Longitudinal results should have the ability to translate findings into information that decision-makers find understandable and compelling. At this time, despite available statistical approaches, results are poorly utilized to change standards of care, mainly due to the lack of standardization and the failure to propose clinical meaningful results.

An ongoing project aims to compare TTD and GLMM using a simulation study [47, 48]. The objective of this project is to propose a standard for longitudinal HRQoL analysis in oncology according to therapeutic situations and cancer sites.

To reach the goal of standardized longitudinal analysis methods for HRQoL, we purport that RECIST criteria for HRQoL regarding TTD are required. We propose the first components of the RECIST criteria here: (1) TTD and TUDD in the adjuvant and advanced/metastatic settings, respectively, with baseline score as a reference, and (2) with the best previous score or the previous score as a reference if RS effect is likely to occur. Further work is needed to achieve a consensus for each cancer setting and tumor site. Moreover, additional investigations are still required regarding the MCID determination to achieve consensus on a definition for MCID.

The TTD approach is already implemented in R software (submitted soon) to allow wider dissemination of these approaches and help move toward the goal of standardization.

At this time, the international ARCAD group (“Aide et Recherche en Cancérologie Digestive”) supports the idea of developing RECIST criteria for HRQoL in colorectal cancer with liver metastasis and pancreatic cancer. Subsequently, HRQoL could then be considered as a co-primary endpoint along with a tumor parameters such as progression-free survival [49]. Future research is warranted on this subject [50]. For example, calculating the number of subjects required for a study with co-primary endpoints is still ongoing.

## Conclusion

The TTD is a didactic and promising approach that we recommend for the longitudinal analysis of HRQoL in oncology, especially because of its capacity to handle RS and to provide results in a format that is familiar to clinicians.

## Electronic supplementary material

Below is the link to the electronic supplementary material.

Supplementary material 1 (PDF 131 kb)

## Abbreviations

CI: Confidence interval
EORTC: European Organisation for Research and Treatment of Cancer
HR: Hazard ratio
HRQoL: Health-related quality of life
GLMM: General linear mixed model
IRT: Item response theory
MCID: Minimal clinically important difference
OS: Overall survival
RS: Response shift
SD: Standard deviation
TTD: Time to deterioration
TUDD: Time until definitive deterioration
