# Supplementary material for: Time to health-related quality of life score deterioration as a modality of longitudinal analysis for health-related quality of life studies in oncology: do we need RECIST for quality of life to achieve standardization?
Source: Qual Life Res. 2013 Nov 26;24(1):5–18. doi: 10.1007/s11136-013-0583-6 (PMC4282717; doi:10.1007/s11136-013-0583-6)
Supplement: Supplementary file 1 — Supplementary material 1 (PDF 131 kb) [file 11136_2013_583_MOESM1_ESM.pdf]

Time to Health-related Quality of Life score deterioration as a modality of longitudinal analysis for health-related quality of life studies in oncology: do we need RECIST for HRQoL to achieve standardization?

Amélie Anota[1,2], Zeinab Hamidou[1,3], Sophie Paget-Bailly[2], Benoist Chibaudel[4,5], Caroline Bascoul-Mollevi[6], Pascal Auquier[1,3], Virginie Westeel[7], Frederic Fiteni[2,8], Christophe Borg[8], Franck Bonnetain[1,2,5]

[1] Quality of Life in oncology clinical research Platform, France

[2] Methodological and Quality of Life in Oncology Unit, EA 3181, University Hospital of Besançon, France

[3] Public health laboratory, EA 3279, Aix-Marseille University, Marseille, France

[4] Medical Oncology Department, CHU Saint-Antoine, Paris, France

[5] Gercor, Clinical Research Group in Oncology

[6] Department of Biostatistics, Regional Cancer Institute, Montpellier, France

[7] Pneumology Department, University Hospital of Besançon, France

[8] Medical Oncology Department, University Hospital of Besançon, France

Corresponding author:

Amélie Anota

Methodological and Quality of Life in Oncology Unit (EA 3181)

University Hospital of Besançon

France

Email: aanota@chu-besancon.fr

Telephone number: +33381218896

Fax number: +33381665299

Additional descriptive results obtained on the breast cancer and pancreatic cancer studies

Table A: Baseline characteristics of patients included in breast cancer study (study #1)

	N	%
**Hospital**		
Dijon	271	71.2
Nancy	74	19.4
Reims	18	4.7
Strasbourg	18	4.7
**Marital status**		
married	281	73.8
not married	90	23.6
unknown	10	2.6
**Inclusion criteria**		
confirmed primitive breast cancer	242	63.5
suspicion of primitive breast cancer	138	36.2
unknown	1	0.3
**Cancer**		
confirmed	340	89.2
not confirmed	38	10.0
unknown	3	0.8
**Lymph node dissection(LND)**		
Axillary LND (ALND)	138	36.2
Sentinel lymph node biopsy (SLNB)	131	34.4
ALND+SLNB	32	8.4
No LND	75	19.7
unknown	5	1.3
**Surgery type**		
mastectomy	124	32.6
no mastectomy	241	63.3
unknown	16	4.1
**Chemotherapy**		
yes	155	40.7
no	218	57.2
unknown	8	2.1
**Radiotherapy**		
yes	254	66.7
no	119	31.2
unknown	8	2.1
**Hormone therapy**		
yes	170	44.6
no	203	53.3
unknown	8	2.1

Table B : Baseline characteristics of patients included in the pancreatic cancer phase II study according to treatment arm (study #2)

	Arm 1 FOLFIRI.3 + gemcitabine (N=49)		Arm 2 gemcitabine alone (N=49)		All patients (N=98)	
	N	%	N	%	N	%
**Sex**						
male	31	63.27	28	57.14	59	60.20
female	18	36.73	21	42.86	39	39.80
**WHO performance status**						
0	16	32.65	16	32.65	32	32.65
1	33	67.35	33	67.35	66	67.35
**Previous surgery**						
no	38	77.55	40	81.63	78	79.59
yes	11	22.45	9	18.37	20	20.41
**Surgery type**						
curative	5	10.20	4	8.16	9	9.18
palliative	5	10.20	5	10.20	10	10.20
not applicable	38	77.55	40	81.63	78	79.59
missing	1	2.04	0	0.00	1	1.02
**Number of metastatic sites**						
1	33	67.35	35	71.43	68	69.39
More than 1	16	32.65	14	28.57	30	30.61
**Previous chemotherapy**						
no	44	89.8	40	81.63	84	85.71
yes	2	4.08	3	6.12	5	5.10
missing	3	6.12	6	12.24	9	9.18
**Primary tumor location**						
head	18	36.70	29	59.20	47	48.00
body	17	34.70	11	22.50	28	28.60
tail	17	34.70	12	24.50	29	29.60
**Sites of metastasis**						
liver	39	79.60	35	71.40	74	75.50
lung	11	22.50	11	22.50	22	22.50
Lymph node	5	10.20	7	14.30	12	12.20
peritoneal	16	33.700	10	20.40	26	26.50
other	3	6.10	2	4.00	5	5.10

Table C: Completion of HRQoL questionnaire at each measurement time by treatment arm for pancreatic cancer study (study #2).

HRQoL assessment	Arm 2 gemcitabine alone N (%)[1]	Arm 1 FOLFIRI.3 + gemcitabine N (%)	All patients N (%)
**baseline**	30/49 (61.2)	34/49 (69.4)	64/98 (65.3)
**cycle 1**	9/21 (42.9)	17/43 (39.5)	33/83 (39.8)
**cycle 2**	2/12 (16.7)	14/32 (43.8)	23/53 (43.4)
**cycle 3**	1/4 (25.0)	9/25 (36.0)	10/37 (27.0)
**cycle 4**	1/4 (25.0)	4/16 (25.0)	5/20 (25.0)
**cycle 5**	0/2 (0.0)	3/12 (25.0)	4/15 (26.7)
**cycle 6**	0/2 (0.0)	2/11 (18.9)	2/9 (22.2)
**cycle 7**	0/1 (0.0)	1/10 (10.0)	1/7 (14.3)
**cycle 8**	0/1 (0.0)	1/10 (10.0)	1/6 (16.7)
**cycle 9**	0/1 (0.0)	1/9 (11.1)	1/6 (16.7)
**cycle 10**	0/0	1/9 (11.1)	1/4 (25.0)

[1] The percentage is given according to potentially responsive patients (excluding death and drop-out)
